# Spontaneous Arachnoid Cyst Rupture with Subdural Hygroma in a Child

**DOI:** 10.1155/2016/6964713

**Published:** 2016-02-18

**Authors:** Muhammad Faisal Khilji, Niranjan Lal Jeswani, Rana Shoaib Hamid, Faisal Al Azri

**Affiliations:** ^1^Department of Emergency Medicine, Sultan Qaboos University Hospital, Al-Khod, P.O. Box 38, 123 Muscat, Oman; ^2^Department of Radiology, Sultan Qaboos University Hospital, Muscat, Oman

## Abstract

Arachnoid cyst of the brain is common in children but its association with spontaneous subdural hygroma is rare. A case of a nine-year-old boy, without any preceding history of trauma, is presented here who came to the emergency department of a tertiary care hospital with complaints of headache, nausea, and vomiting for the last two weeks but more for the last two days. Examination showed a young, fully conscious oriented boy with positive Cushing's reflex and papilledema of left eye. MRI (magnetic resonance imaging) of the brain showed left temporal extra-axial cystic lesion of 5.40 × 4.10 cm in size, representing arachnoid cyst, with bilateral frontoparietal subdural hygromas. Cyst was partially drained through left temporal craniectomy and subdural hygromas were drained through bilateral frontal burr holes. Postoperatively the child recovered uneventfully and was discharged on the seventh postoperative day. Histopathology proves it to be arachnoid cyst of the brain with subdural CSF (cerebrospinal fluid) collection or hygroma.

## 1. Introduction

Arachnoid cysts are common childhood developmental anomalies, but their association with spontaneous subdural hygroma is rare [1–3]. They are benign lesions and may be associated with complications such as subdural hematoma, subdural hygroma, and intracystic hemorrhage. Minor head injury is known to result in subdural hygroma or hematoma due to rupture of arachnoid cyst; however spontaneous rupture of arachnoid cyst may occur rarely [3]. Most of these cysts are in middle cranial fossa where they are likely to be symptomatic if they are large. Chances of expansion are greatest in middle cranial fossa [1]. They are more common in males as compared to females [3, 4]. In children they are frequently associated with seizures and cranial deformity [1, 5, 6].

## 2. Case Report

**Figure 1:**
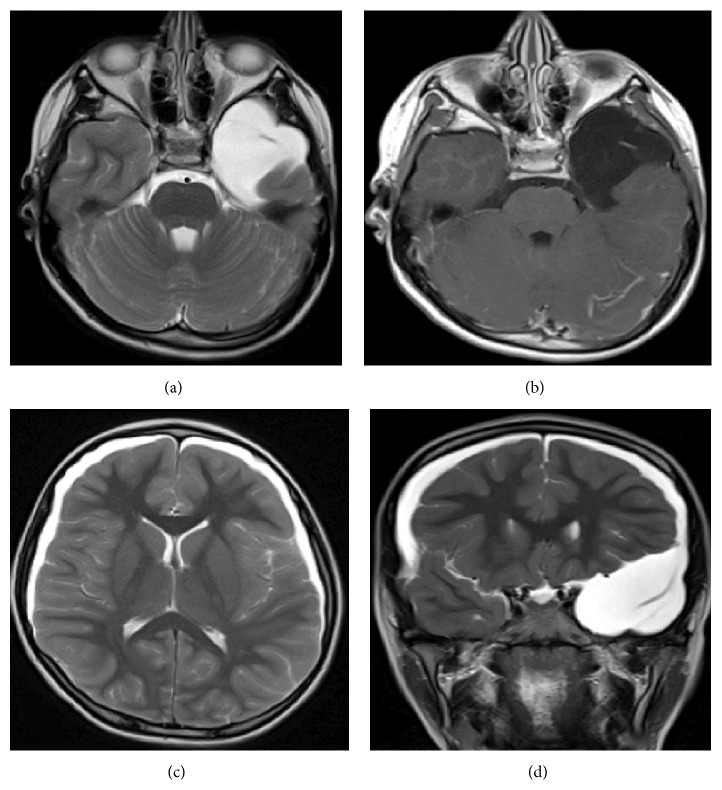
MRI brain axial T2 and T1 weighted postcontrast images (a) and (b) showing an extra-axial fluid signal intensity lesion in the left temporal region without any solid component or abnormal enhancement. Axial and coronal T2 weighted images show bilateral frontoparietal crescent shaped subdural fluid collections.

We present a case of a nine-year-old, previously healthy boy, presenting to the emergency department of a tertiary care hospital with complaints of severe frontal headache for the last two weeks but more for the last two days. Headache was associated with nausea, vomiting, photophobia, and pain in both eyes. There was no history of fever, jerking movements, or trauma. The child had no past medical or surgical history. He achieved all developmental milestones normally and was doing well at school. His examination revealed a fully conscious and oriented child with a heart rate of 77 per minute and blood pressure of 159/112 mm Hg. Power was 5/5 on both sides, all cranial nerves were intact, and no sensory loss was observed. There were no meningeal signs but there was right side papilledema. The rest of the exam was normal. His blood investigations, including blood glucose, full blood count, serum creatinine, and electrolytes, were normal. MRI of the brain was done, after initial symptomatic management. MRI showed left temporal, extra-axial collection measuring 5.40 × 4.10 cm of CSF-like intensity compatible with arachnoid cyst and bilateral frontoparietal subdural enlargement compatible with subdural hygroma (Figure 1).

Within an hour of ED admission the child's frequency of vomiting increased along with mild drowsiness. At that point Cushing's reflex was positive with heart rate was 64/minute and blood pressure of 177/110 mm Hg. The child was referred to the neurosurgery team and the subdural hygromas were drained through bilateral frontal burr holes. Left temporal arachnoid cyst was partially drained through left temporal craniotomy. A left subdural-peritoneal shunt was placed for drainage of any future collection. Drained CSF was xanthochromic and negative for malignant cells. CSF culture showed no growth. Histopathology of cyst confirmed radiological diagnosis of arachnoid cyst. Final diagnosis of left temporal arachnoid cyst with spontaneous subdural hygroma was made as the child presented symptomatically in the absence of any history of trauma. Postoperatively the child recovered uneventfully and was discharged on the 7th postoperative day. Any medium term CT scan was not performed.

## 3. Discussion

Arachnoid cysts are CSF collections between the two layers of arachnoid membrane and are mainly benign lesions. About 1% of intracranial space occupying lesions is arachnoid cysts. They usually grow slowly and occasionally disappear without treatment [1, 7]. Arachnoid cysts complicated, with cystic expansion, intracystic hemorrhage, subdural hematoma, and subdural hygroma, are usually symptomatic. Their association with subdural hygroma is rare [8–10]. In 1924 Naffziger postulated, for the first time, the possible mechanism of subdural hygroma formation [11], according to which a tear in the arachnoid membrane resulting in one-way valve mechanism with accumulation of CSF is thought to be responsible for subdural hygromas [12]. Tears of outer cyst membrane are the main cause of subdural hygroma in arachnoid cyst patients. The rupture is either traumatic, due to surgical manipulation, or rarely spontaneous [13]. Raised intracranial pressure with Valsalva maneuver is another possible cause of cyst rupture leading to hygroma. Most of the cases present with symptoms of raised intracranial pressure like nausea, vomiting, headache, and rarely diplopia from VI nerve palsy. Parsch et al. reported subdural hemorrhage in 2.43% and subdural hygroma in 0.46% of patients with arachnoid cyst reported on MRI [6]. Mild head injury is the most common cause of arachnoid cyst rupture; however rarely do they rupture spontaneously [8]. Gradual increase in size of hygroma is due to the continuous transudation of cerebrospinal fluid in the ruptured cyst. CSF itself is less expansive and compressive than blood due to its low osmotic and hydrostatic pressure [8]. Patients with arachnoid cyst may have complications by subdural hematoma as they are predisposed to it [8]. Different treatment options of arachnoid cyst are controversial. Surgical treatment of symptomatic cysts is generally acceptable. Craniotomy and fenestration of cyst, subdural evacuation, and CSF derivation are procedures performed in arachnoid cyst rupture depending upon the size, location, and clinical presentation [1]. CSF derivation is the preferred procedure in patients with raised intracranial pressure [3]. Di Rocco et al. demonstrated the usefulness of prolonging preoperative intracranial pressure recording [14]. Craniotomy and fenestration is adopted to treat well-circumscribed cysts. Cyst either decreases or disappears after subdural evacuation [13]. Prophylactic surgical treatment is not recommended.

## 4. Conclusion

Spontaneous subdural hygroma should be suspected in symptomatic arachnoid cyst patients without any history of head injury. Symptomatic hygroma is not an absolute indication of surgical treatment; however most of such patients need surgical treatment.

## References

[1] Rajesh A., Bramhaprasad V., Purohit A. K. (2012). Traumatic rupture of arachnoid cyst with subdural hygroma. *Journal of Pediatric Neurosciences*.

[2] Donaldson J. W., Edwards-Brown M., Luerssen T. G. (2000). Arachnoid cyst rupture with concurrent subdural hygroma. *Pediatric Neurosurgery*.

[3] Albuquerque F. C., Giannotta S. L. (1997). Arachnoid cyst rupture producing subdural hygroma and intracranial hypertension: case reports. *Neurosurgery*.

[4] Sener R. N. (1997). Arachnoid cysts associated with post-traumatic and spontaneous rupture into the subdural space. *Computerized Medical Imaging and Graphics*.

[5] Choux M., Raybaud C., Pinsard N., Hassoun J., Gambarelli D. (1978). Intracranial supratentorial cysts in children excluding tumor and parasitic cysts. *Child's Brain*.

[6] Parsch C. S.,  Krauss J., Hofmann E., Meixensberger J., Roosen K. (1997). Arachnoid cysts associated with subdural hematomas and hygromas: analysis of 16 cases, long-term follow-up, and review of the literature. *Neurosurgery*.

[7] Dodd R. L., Barnes P. D., Huhn S. L. (2002). Spontaneous resolution of a prepontine arachnoid cyst: case report and review of the literature. *Pediatric Neurosurgery*.

[8] Poirrier A.-L. M. L., Ngosso-Tetanye I., Mouchamps M., Misson J.-P. (2004). Spontaneous arachnoid cyst rupture in a previously asymptomatic child: a case report. *European Journal of Paediatric Neurology*.

[9] Cakir E., Kayhankuzeyli, Sayin O. C., Peksoylu B., Karaarslan G. (2004). Arachnoid cyst rupture with subdural hygroma: case report and literature review. *Neurocirugia*.

[10] Offiah C., Forbes W. S. C., Thorne J. (2006). Non-haemorrhagic subdural collection complicating rupture of a middle cranial fossa arachnoid cyst. *British Journal of Radiology*.

[11] Naffziger H. C. (1924). Subdural fluid accumulations following head injury. *The Journal of the American Medical Association*.

[12] Çokluk C., Şenel A., Çelik F., Ergür H. (2003). Spontaneous disappearance of two asymptomatic arachnoid cysts in two different locations. *Minimally Invasive Neurosurgery*.

[13] Mori K., Yamamoto T., Horinaka N., Maeda M. (2002). Arachnoid cyst is a risk factor for chronic subdural hematoma in Juveniles: twelve cases of chronic subdural hematoma associated with arachnoid cyst. *Journal of Neurotrauma*.

[14] Di Rocco C., Tamburrini G., Caldarelli M., Velardi F., Santini P. (2003). Prolonged ICP monitoring in Sylvian arachnoid cysts. *Surgical Neurology*.

